# Aerobic glycolysis-driven lactate accumulation and acidic tumor microenvironment in hepatocellular carcinoma: a vicious cycle promoting tumor progression

**DOI:** 10.3389/fphar.2026.1704729

**Published:** 2026-05-08

**Authors:** Mengzhuo Liu, Junzhe Jiao, Haibo Zhang, Zhanjie Chang, Ning Wang, Xi Guan, Ruijuan Yan, Qian Huang, Qi Xi, Xiaojiaoyang Li, Jianfeng Bao, Shuguang Yan, Jingtao Li

**Affiliations:** 1 Shaanxi University of Chinese Medicine, Xianyang, China; 2 Departments of Infectious Disease, The Affiliated Hospital of Shaanxi University of Chinese Medicine, Xianyang, China; 3 College of Basic Medicine, Shaanxi University of Chinese Medicine, Xianyang, China; 4 Department of Pathology, Shaanxi University of Chinese Medicine, Xianyang, China; 5 Department of Warm Disease, Shaanxi University of Chinese Medicine, Xianyang, China; 6 Beijing University of Chinese Medicine, Beijing, China; 7 The Hangzhou Xixi Hospital Affiliated to the Zhejiang Chinese Medical University, Hangzhou, China; 8 Key Laboratory of Gastrointestinal Diseases and Prescriptions in Shaanxi Province, Shaanxi University of Chinese Medicine, Xianyang, China

**Keywords:** acidic tumor microenvironment, aerobic glycolysis, hepatocellular carcinoma, lactate accumulation, targeted therapeutic strategies

## Abstract

**Background:**

Hepatocellular carcinoma (HCC) is a highly lethal malignancy with rising global incidence. Aberrantly activated aerobic glycolysis is a hallmark of HCC, driving excessive lactate production and the formation of an acidic tumor microenvironment (TME) that promotes malignant progression. Elucidating the underlying mechanisms of this metabolic axis is essential for developing targeted therapeutic strategies.

**Objective:**

This review systematically examines the role of the aerobic glycolysis-lactate-acidic TME axis in HCC progression, focusing on its molecular basis, multifaceted functions of lactate, and therapeutic implications.

**Key Findings:**

HCC progression is driven by a coordinated “production-efflux” mechanism involving glycolytic rate-limiting enzymes (such as HK2, PKM2, LDH) and the lactate transporter such as MCT4, which together facilitate lactate accumulation and TME acidification. Lactate exerts pleiotropic effects within the TME, including metabolic signaling via GPR81 and ASICs, epigenetic regulation through histone lactylation, and inter-organ crosstalk via the gut–liver axis. Current therapeutic strategies targeting GLUTs, glycolytic enzymes, LDH, and MCT4 are reviewed, along with approaches for direct TME alkalinization and combination regimens with targeted agents. Building upon this landscape, the present review is the first to systematically integrate emerging mechanisms such as histone lactylation and the gut-liver axis, and to explore the synergistic potential of combining metabolic inhibitors with immunotherapy, thereby offering a distinct framework that moves beyond traditional glycolysis-centric narratives in HCC.

**Challenges and Perspectives:**

Despite the substantial potential of targeting this metabolic axis, a considerable gap remains between preclinical findings and clinical translation. The pronounced heterogeneity of HCC, along with metabolic plasticity and insufficient drug selectivity, poses major challenges. Future efforts should prioritize the development of highly selective inhibitors and the optimization of combination therapies based on metabolic stratification.

## 1 Introduction

According to recent epidemiological data, the incidence of primary liver cancer (PLC) has demonstrated a consistent upward trend, and it now ranks as the sixth most prevalent malignancy worldwide. In China, PLC-associated morbidity and mortality remain alarmingly high, with the disease ranking as the fifth and second leading causes of cancer-related morbidity and mortality, respectively (National Health Commission, 2024). Hepatocellular carcinoma (HCC), accounting for 70%–85% of PLC cases, is characterized by insidious onset, limited therapeutic efficacy, and poor prognosis. Despite advances in early diagnosis and treatment, the rates of recurrence and mortality associated with HCC remain unacceptably high. Therefore, elucidating the molecular pathogenesis and key regulatory mechanisms underlying HCC is essential for the development of precise therapeutic strategies aimed at improving clinical outcomes.

Over the past decades, research on HCC pathogenesis has predominantly focused on the precise identification and targeting of specific molecular structures and oncogenic genes in tumor cells. More recently, accumulating evidence has shifted attention toward aberrant tumor metabolism, with metabolic reprogramming emerging as a hallmark of HCC development. Among the various facets of metabolic reprogramming, aerobic glycolysis has become a prominent area of investigation. Highly activated aerobic glycolysis in hepatocytes results in excessive glucose consumption and lactate accumulation, contributing to the formation of an acidic tumor microenvironment (TME) that favors tumor survival. This review provides a comprehensive overview of the role of the lactate-enriched acidic TME in HCC progression, encompassing local invasion, proliferation, metastasis, immune evasion, and therapeutic resistance. In addition, we explore novel diagnostic and therapeutic strategies targeting this metabolic axis.

## 2 Aerobic glycolysis in HCC: core enzymes and pathways

Aerobic glycolysis serves as a cornerstone of tumor metabolism, fueling malignant proliferation while establishing an acidic TME that supports tumor survival. Under normal conditions, adenosine triphosphate (ATP) is generated primarily via oxidative phosphorylation (approximately 90%) and glycolysis (approximately 10%); in tumor cells, however, this ratio shifts significantly to roughly 40% and 60%, respectively (Feng et al., 2020). This metabolic reprogramming drives tumor cells to preferentially utilize glycolysis even under normoxic conditions—a phenomenon known as the Warburg effect (Yu et al., 2018)—resulting in glucose depletion and substantial lactate production. Given the heightened demands of angiogenesis, local invasion, and rapid proliferation, tumor cells frequently experience nutrient scarcity and therefore must upregulate aerobic glycolysis to satisfy their bioenergetic requirements (Yang F. et al., 2023). This adaptation not only enhances ATP synthesis and glycolytic flux but also supplies downstream biosynthetic precursors essential for tumor cell proliferation (Ganapathy-Kanniappan, 2017). Although the Warburg effect received limited attention for decades following its initial proposal and was long considered a minor facet of tumor metabolism, accumulating evidence over the past 2 decades has established its central role in tumorigenesis and progression, with its fundamental mechanisms being progressively elucidated across diverse tumor types.

**FIGURE 1:**
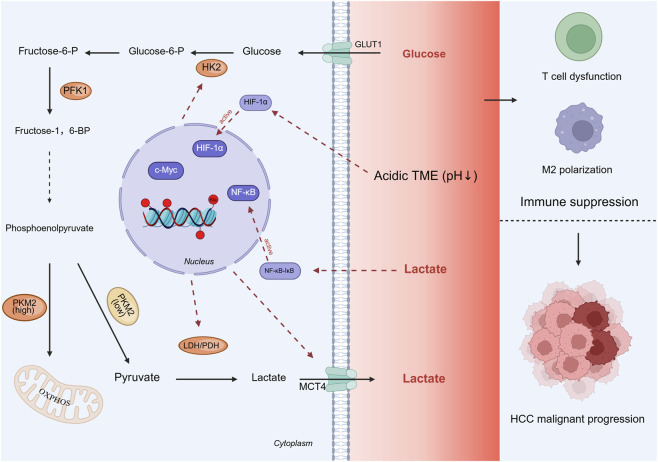
Purple-labeled upstream regulators (HIF-1α, c-Myc, NF-κB) promote the expression of orange-labeled glycolytic enzymes (HK2, PFK1, PKM2, LDHA) and green-labeled transporters (GLUT1, MCT4). Glucose is metabolized to lactate via this pathway, and lactate is effluxed to the extracellular space by MCT4, forming an acidic tumor microenvironment (TME, indicated by a red gradient). The acidic TME further activates a feedback loop (red dashed arrows), which on one hand activates HIF-1α and NF-κB, and on the other hand induces histone lysine lactylation (Kla, red) and suppresses immune cell functions (M2 polarization and T cell dysfunction). These mechanisms, together with other effects, synergistically promote malignant progression of HCC. See text for details. Permission to reuse and Copyright. Created in BioRender. https://BioRender.com/l33n503.

Upon activation of aerobic glycolysis, glucose is first transported across the cell membrane via glucose transporters (GLUTs) and then phosphorylated to glucose-6-phosphate (Glucose-6-P) by hexokinase 2 (HK2). In the glycolytic pathway, glucose-6-P is subsequently converted to fructose-6-phosphate (Fructose-6-P) and further metabolized to fructose-1,6-bisphosphate (Fructose-1,6-BP) by phosphofructokinase-1 (PFK1). Following a series of enzymatic reactions, phosphoenolpyruvate is generated as a key intermediate at the junction of glycolysis and gluconeogenesis. The subsequent fate of phosphoenolpyruvate is determined by the activity state of pyruvate kinase M2 (PKM2). In normal cells, PKM2 predominantly exists as a highly active tetramer, directing phosphoenolpyruvate toward mitochondrial entry and subsequent tricarboxylic acid cycle engagement to support routine cellular functions. In contrast, tumor cells often exhibit PKM2 in a low-activity dimeric form, which catalyzes the conversion of phosphoenolpyruvate to pyruvate while simultaneously causing upstream metabolite accumulation—a metabolic shift that favors aerobic glycolysis. Pyruvate is then catalyzed to lactate by lactate dehydrogenase (LDH), and the resulting lactate is effluxed to the extracellular compartment via monocarboxylate transporters (MCTs), thereby establishing a coordinated “production-export” axis Figure 1.

## 3 Lactate and acidic TME: biological functions

The aforementioned aerobic glycolysis is highly activated in tumor cells, leading to increased extracellular lactate content and further formation of acidic TME. For many years, lactate has often been considered a metabolic waste, but nowadays, the prominent role of lactate and acidic TME for HCC is realized. Since the development, proliferation, metastasis, immune escape, and resistance of HCC mainly occur within this acidic TME matrix and break down or destroy the standard extracellular environment matrix, all biological processes of HCC are greatly affected by it. The TME is intricate and includes a material-based environment that supports the survival of tumor cells in addition to the presence of various intercellular biological interactions.

Lactate tends to exist in the body as an ionized form that includes lactate and H^+^ components, and the tumor-promoting effects of lactate depend on both. The acidic TME outside tumor cells is enriched with H+, causing a transmembrane electrochemical H^+^ gradient (also known as proton motive force) on the plasma membrane of tumor cells consistent with that of normal cells, which allows essential nutrients that maintain the physiological function of normal cells, such as amino acids, iron ions, folic acid, etc., to provide energy for tumor cells by actively entering them (Brown and Ganapathy, 2020). This process creates a situation in which the tumor cells are enriched with high-quality nutrients while the normal cells remain in a lousy situation in which nutrients are lacking. The final stage of tumor development also includes a critical step in which tumor cells survive and proliferate, relying on aberrant energy metabolism that allows them to escape apoptotic process (Chang et al., 2021).

Lactate is indispensable in tumor development as it upregulates cancer gene expression, induces angiogenesis, and promotes local infiltration and distant metastasis of tumor cells to create a suitable pre-metastatic ecological niche for tumor cells to survive (Ippolito et al., 2019; Chen S. et al., 2024). The extracellular environment of a healthy organism is primarily alkaline, with a pH range of 7.2–7.5 in most cases, whereas the abnormal TME is acidic, with a pH value of approximately 6.5–6.9 (Choi et al., 2013). Excessive lactate maintains low pH homeostasis and acidic TME, which affects the activities of various enzymes and induces multiple immune cells to jointly construct an immunosuppressive network, contributing to the immune escape of tumor cells (Choi et al., 2013; Ganapathy-Kanniappan, 2017; Wang et al., 2020). The metastasis, recurrence, and poor prognosis of various types of tumors, such as HCC and cervical cancer (Roth and Decaens, 2017), have a direct positive relationship with the extracellular acidic TME.

## 4 Lactate and acidic TME: molecular mechanisms

### 4.1 Conventional mechanisms

The biomolecules most strongly associated with lactate production and transport during glycolysis were LDH and MCTs, respectively.LDH, as a glycolytic enzyme that mediates the redox reaction between pyruvate and lactate, consists of three subunits, LDHA, LDHB, and LDHC, and can constitute six tetrameric isozymes. LDH is widely present in human tissues and is particularly expressed in large quantities in tumor cells, which have a much higher affinity for pyruvate (Doherty and Cleveland, 2013). Under this effect, large amounts of lactate are continuously produced in tumor cells (Certo et al., 2021). Studies have shown that double knockdown of LDHA and LDHB genes in two tumor cell lines, human intestinal adenocarcinoma, and mouse melanoma cells, completely inhibited LDH activity and lactate secretion and led to a genetic blockade of tumor cell growth, proliferation, and metastasis (Ždralević et al., 2018). Moreover, high expression of LDHA has been found in different types of tumors, and is highly correlated with poor overall survival (Lu et al., 2015).

MCTs function as lactate transport proteins in tumor cells, mediating the transmembrane transport of lactate. Among these, MCT1 and MCT4 are primarily implicated in HCC, yet they differ in substrate directionality, tissue distribution, and functional roles. MCTs facilitate the efflux of accumulated lactate from HCC cells into the extracellular matrix, thereby preventing intracellular acidosis and contributing to the establishment and maintenance of an acidic TME. MCT1 is widely expressed in both normal and neoplastic tissues and is capable of independently controlling lactate influx and efflux depending on the transmembrane concentration gradient, thus enabling bidirectional lactate transport. In contrast, MCT4 is selectively overexpressed in hypoxic and glycolytic tumor cells and plays a dominant role in establishing the acidic TME and promoting HCC progression (Felmlee et al., 2020). In highly glycolytic HCC, excessive intracellular lactate saturates MCT1, leading to compensatory upregulation of MCT4 to facilitate lactate efflux and avert intracellular acidosis (Payen et al., 2020). Functional studies have demonstrated that blocking MCT1 expression in HCC cell lines suppresses lactate efflux and glucose metabolism, thereby inhibiting HCC cell proliferation (Huang et al., 2014). Similarly, MCT4 expression is upregulated in HCC cells and promotes tumor metastasis and proliferation (Gao et al., 2015). Furthermore, knockout of *SLC16A3*, the gene encoding MCT4, reduces extracellular lactate concentration, alleviates hypoxia, and induces ferroptosis in HCC (Shen et al., 2024), further supporting MCT4 as a potential therapeutic target. Collectively, these findings underscore that MCT4, rather than MCT1, is a key driver of lactate efflux and acidic TME formation in HCC. High expression of MCT1 and MCT4 in tumor cells has also been associated with poor prognosis in various other cancers, including gastric cancer (GC) (Yan et al., 2014), colorectal cancer (Pereira-Vieira et al., 2019), non-small cell lung cancer (NSCLC) (Eilertsen et al., 2014), and breast cancer (BC) (Yuan et al., 2021), indicating a broader role for these transporters in tumor metabolism.

In tumor cells characterized by aberrantly activated aerobic glycolysis, rate-limiting enzymes orchestrate the stepwise catalytic conversion of glucose to pyruvate, while LDHA subsequently converts pyruvate to lactate. Together, these enzymes synergistically potentiate the lactate-producing capacity within tumor cells. Concurrently, MCT4 functioning as the principal efflux transporter for lactate, efficiently extrudes accumulated intracellular lactate into the extracellular microenvironment (Jinmei and Zhizhong, 2020). This concerted “production-efflux” cooperative mechanism confers dual advantages to tumor cells: on one hand, the elevated glycolytic flux facilitates rapid ATP generation and supplies metabolic intermediates, thereby meeting the bioenergetic and biosynthetic demands required for rapid proliferation; on the other hand, MCT4-mediated lactate efflux effectively averts intracellular acidosis, which would otherwise impair metabolic enzyme activity and cell viability, while concurrently enabling lactate to act as a signaling molecule within the TME, contributing to the establishment of an acidic milieu and modulation of immune cell functions. Hence, this cooperative axis not only epitomizes the hallmark of aberrantly activated aerobic glycolysis in HCC but also constitutes a pivotal pathological mechanism within its metabolic reprogramming, orchestrating energy production, detoxification, and microenvironmental remodeling.

In addition to LDH and MCTs, lactate accumulation associated with aerobic glycolysis is co-regulated by multiple classical biomolecules and signaling pathways.

Mitochondrial DNA (mtDNA) (Kim et al., 2022; Mahmood et al., 2024) is the genetic material in mitochondria that encodes oxidative phosphorylation and metabolic homeostasis (Mahmood et al., 2024). The probability of mtDNA mutation is exceptionally high in the genomes of tumor cells. It has been demonstrated in a mouse model of melanoma that mutated mtDNA can substantially promote aerobic glycolysis metabolism and remodel the TME, leading to a decreased immune response to tumors in the form of neutrophil loss (Kim et al., 2022). Mammalian target of rapamycin (mTOR) (Zhang et al., 2022) is another regulator associated with aerobic glycolysis in HCC. mTOR-NEAT1 signaling, as example, is observed in activated HCC cells, which upregulates lncRNA transcription and the expression of related glycolysis rate-limiting enzymes, leading to high glycolysis levels, promoting lactate production and accelerating HCC cell proliferation (Zhang et al., 2022).

In addition to extensive lactate accumulation, hypoxia constitutes another hallmark feature of the TME. Within the TME, hypoxia induces the binding of hypoxia-inducible factor-1α (HIF-1α) to its cognate response elements, leading to hyperactivation of glycolysis and stimulation of angiogenesis, which in turn exacerbates hypoxia. Concurrently, large amounts of lactate released by tumor cells via glycolysis also promote angiogenesis. Thus, aerobic glycolysis, lactate, angiogenesis, and hypoxia form a positive feedback loop that progressively drives tumor progression. This self-amplifying feedback loop not only drives tumor progression but also actively establishes an immunosuppressive microenvironment that disrupts the liver’s intrinsic immune surveillance.

The liver possesses distinctive immunological properties; nevertheless, the incidence of HCC remains high. This paradox is largely attributable to the acidic TME, which suppresses both local and systemic immunity (Piñeiro Fernández et al., 2019). In HCC, the accumulation of lactate and protons results in a persistently low extracellular pH, which directly compromises immune cell function. Acidic conditions inhibit the proliferation and cytotoxic activity of CD8^+^ T cells and natural killer cells, while promoting apoptosis of effector lymphocytes (Hosonuma and Yoshimura, 2023; Rahman et al., 2024). Simultaneously, elevated lactate concentrations impair the metabolic fitness of immune cells by competing with glucose for cellular uptake and glycolysis, thereby limiting their energy supply and antitumor capacity (Jiang et al., 2024). In contrast to effector cells, regulatory T cells (Treg cells) exhibit greater resistance to acidity and may even enhance their immunosuppressive functions within the acidic TME (Rahman et al., 2024). These well-established mechanisms—pH-mediated cytotoxicity, metabolic competition, and the relative resistance of Tregs cells—collectively establish a broad immunosuppressive barrier in the HCC TME, independent of specific receptors or epigenetic modifications (Peng et al., 2024).

### 4.2 Novel mechanisms

In recent years, beyond these well-established mechanisms, emerging advances have been made regarding the biological processes of lactylation associated with aerobic glycolysis in the TME, including histone lysine lactylation (Kla), G-protein-coupled receptor 81 (GPR81), acid-sensing ion channels (ASICs), the gut-liver axis, and other related mechanisms Table 1.

**TABLE 1:** Key signaling pathways and transcription factors regulating aerobic glycolysis in HCC.

Regulator	Type	Target glycolytic enzymes/transporters	Expression trend	References
PI3K/Akt	Signaling pathway	GLUTs, HK2, PFK2, AMPK	Upregulated	Ward and Thompson (2012), Guerrero-Zotano et al. (2016), Feng et al. (2020), Zhang et al. (2024b)
AMPK	Signaling pathway	GLUTs, PFK2	Downregulated	Gao et al. (2019), Ren and Shen (2019), Feng et al. (2020)
mTOR	Signaling pathway	PKM2, GLUT1, HK2, LDHA	Upregulated	Zhang et al. (2022); Marques-Ramos and Cervantes (2023)
NF-κB	Signaling pathway/Transcription factor	GLUT1, HK2, LDHA	Upregulated	Chen et al. (2022); Guo et al. (2022); Raghav and Jeong (2024)
HIF-1α	Transcription factor	GLUT, HK2, MCT4, PKM, Aldolase, Fructose-Bisphosphate A, Enolase 2	Upregulated	Bao et al. (2021)
RFX6	Transcription factor	Phosphoglycerate mutase 1	Upregulated	Qiu et al. (2023)
c-Myc	Transcription factor	GLUTs, HK2, PKM2, LDH, PFK	Upregulated	Hsieh et al. (2015)
ACE2	Membrane-bound enzyme	SLC2A1, HK2, Alpha-enolase, PFKL, LDHA, PDK1	Downregulated	Dong et al. (2023)
METTL5	Methyltransferase	GLUTs, HK2, PFK2, AMPK	Upregulated	Van Tran et al. (2019); Chen and Wong (2020); Xia et al. (2023)
GTPBP4	GTP-binding protein	PKM2	Upregulated	Zhou et al. (2022)
mtDNA	Mitochondrial DNA	GAPDH, MDH1, LDHA	Upregulated	Kim et al. (2022); Mahmood et al. (2024)

In 2019, Kla was identified as a lactate-driven epigenetic modification that links glycolysis to gene expression (Zhang et al., 2019). This modification provides a mechanistic explanation for how lactate drives tumor progression by promoting angiogenesis, proliferation, metastasis, and immune escape. Kla induces M2 polarization of macrophages and enhances the transcription of immunosuppressive genes, including programmed death-ligand 1 (PD-L1). Mechanistically, lactate is converted to lactyl-coenzyme A and transferred to histone lysine residues by the acetyltransferase p300/CBP, leading to PD-L1 upregulation. Moreover, lactate directly lactylates PD-L1 at lysine 162, which prevents its ubiquitination by the E3 ligase STUB1 and stabilizes PD-L1 on the tumor cell membrane (Yang Z. et al., 2023). Subsequent studies have further expanded the scope of lactylation and confirmed that Kla originates from cellular lactate, with Kla levels positively correlating with lactate concentrations, thereby establishing a positive feedback loop that amplifies immunosuppressive signals (Zhang et al., 2019). In hepatitis B virus-associated HCC, high Kla levels correlate with increased PD-L1 expression, T-cell dysfunction, and poor prognosis (Yang Z. et al., 2023). Pharmacological inhibition of LDHA or blockade of PD-L1 partially restores T-cell function, indicating that the lactylation/PD-L1 axis represents a promising therapeutic target for overcoming immunotherapy resistance (Angelin et al., 2017; Hao et al., 2024; Tong et al., 2024).

In addition to its role as an energy-rich metabolite, lactate functions as an important signaling molecule. GPR81, a typical lactate receptor, is expressed on the surface of HCC cells. During tumor cell proliferation, lactate acts as an agonist of GPR81, activating the receptor via both autocrine and paracrine pathways (Brown and Ganapathy, 2020). Upon lactate binding, GPR81 triggers downstream signaling cascades that upregulate PD-L1 transcription through promoter activation and transcription factor engagement (Feng et al., 2017). GPR81 signaling further promotes angiogenesis, local invasion, and immune evasion (Brown and Ganapathy, 2020). Thus, GPR81 constitutes an additional route through which lactate directly controls PD-L1-mediated immunosuppression.

ASICs are sensitive protein receptors that sense cellular acidity and alkalinity changes activated by decreased pH and increased H^+^ in the extracellular environment. They have a wide range of functions; for example, they are involved in inflammatory responses, acidosis, and tumor proliferation and metastasis in a variety of diseases in the body. The expression of ASIC1a can also be detected in the tumor cells of patients with hepatitis B virus-associated HCC, and silencing of ASIC1a reveals that the invasive metastatic ability of HCC cells is inhibited (Jin et al., 2015).

The gut-liver axis and gut microbiota, along with their metabolic products, play a pivotal role in maintaining acid-base homeostasis within the body. Analysis of gut microbiota in HCC patients has revealed that diverse bacterial flora, such as *Clostridium*, can modulate the TME, proving highly significant in the development of HCC (Zuo et al., 2025). Hence, we tentatively propose that high-flux aerobic glycolysis and the formation of acidic TME are likewise directly or indirectly regulated by them.

In both *in vivo* and *in vitro* experiments using a mouse xenograft HCC model, sodium butyrate (NaBu), a metabolic product of gut microbiota, was observed to inhibit activation of the c-Myc/HK2 signaling pathway. It affects glucose uptake and lactate production, directly suppressing aerobic glycolysis. Furthermore, certain microbial metabolites indirectly influence aerobic glycolysis through immunomodulation, a process closely linked to immune microenvironment remodeling. In the MASH-HCC model, high-fat diet-induced dysbiosis impedes gluconeogenesis, promoting cellular reorientation towards glycolysis and increasing lactate accumulation34. Further studies confirmed that this model also alters short-chain fatty acid ratios and induces Treg expansion, while the high-lactate environment further inhibits CD8^+^ T cell activity. The gut microbial metabolite d-lactate can regulate tumor-associated macrophage (TAM) polarization towards the M1 phenotype, remodeling the immune microenvironment and indirectly influencing HCC cell metabolic states (Ju et al., 2025). In summary, the gut microbiota can directly regulate aerobic glycolysis in tumor cells via its metabolites, whilst this metabolic reprogramming reciprocally shapes the immune microenvironment. This forms a positive-feedback “microbiota-metabolism-immunity” triple-regulatory network.

Most studies on aerobic glycolysis have focused on the initial initiation of this biological process, such as the positive promotion of aerobic glycolysis by certain types of biological proteins, chemokines, or signaling pathways. However, a recent study on angiotensin-converting enzyme 2 (ACE2) (Dong et al., 2023) revealed a negative feedback regulation of aerobic glycolysis by ACE2 in HCC. Preliminary investigations have revealed that ACE2 expression is downregulated in HCC and correlates with poor prognosis. Further studies revealed that ACE2 can inhibit glycolytic flux by activating SHP2 phosphorylation and inhibiting HIF-1α. In addition, ACE2 overexpression in a patient xenograft model significantly delayed the growth of HCC tumors (Dong et al., 2023). The above experiments have confirmed the strong association between various biosignals and lactate enrichment after high-flux aerobic glycolysis in different ways and have provided novel perspectives and research potentials for the metabolism and biology of HCC and other types of cancers.

## 5 Therapeutic targets and clinical advances

Up until now, there have been many treatment options for HCC, mainly guided by the Barcelona Clinic Liver Cancer staging system. Early-stage HCC patients can choose surgical resection, local radiofrequency ablation, liver transplantation, etc.,; intermediate and late-stage patients can choose transcatheter arterial chemoembolization (TACE) and hepatic artery infusion chemotherapy (HAIC); most of the late-stage patients are treated with systemic therapy such as oral targeted drugs like sorafenib, lenvatinib, etc., or injections of immune checkpoint inhibitors like karelizumab, bevacizumab, etc.,; moreover, patients with HCC whose pathology is HBV or HCV infection should be concurrently treated with additional antiviral drugs or injections of interferon (Zhou et al., 2023). Most regimens aim to improve patients’ overall survival rate, prolong survival time and reduce tumor recurrence, and so on. However, it should not be overlooked that HCC is relatively insensitive to radiotherapy and chemotherapy, and the quality of life of patients in the advanced stages of the disease declines sharply. As patient’ needs for survival time and quality of life increase, direct or adjuvant pharmacological treatments for HCC have been extensively developed in recent years, and studies targeting aerobic glycolysis have also become increasingly abundant Table 2.

**TABLE 2:** Therapeutic strategies targeting aerobic glycolysis and lactate metabolism.

Agent	Target	Mechanism	Clinical status	Cancer type	Key limitations	References
Everolimus	mTOR	Inhibits mTORC1, reducing HIF-1α and c-Myc expression and downstream glycolytic enzymes	Approved (other cancers); Phase III failed (HCC)	RCC, Pancreatic neuroendocrine tumor	HCC population: negative OS	Azar et al. (2025); Cros et al. (2025)
Temsirolimus	mTOR	Inhibits mTORC1 signaling, indirectly suppressing glycolysis	Approved (other cancers); Phase III failed (HCC)	RCC	HCC population: negative OS	Ma et al. (2009); Gu et al. (2022)
Cagliflozin	SGLT2	Inhibits SGLT2, reducing glucose uptake and glycolysis in cancer cells	Approved (non-oncology indication); Preclinical, phase II exploratory ongoing (HCC)	PLC	Limited patient population, lack of prospective RCT validation	Obara et al. (2017)
Mitapivat	PKM2	Converting low-activity PKM2 dimers into high-activity tetramers	Approved (other cancers); preclinical (HCC)	HCC, BC, leukaemia, Lymphoma	Hepatocellular injury, risk of acute hemolysis	Matte et al. (2023)
Kaempferol	HK2, aldolase A	Inhibiting HK2 and aldolase A; enhancing oxidative phosphorylation	Phase III completed (non-oncology indication); preclinical (HCC)	Melanoma, CRC	Extremely low bioavailability, rapid metabolism	Wu et al. (2021); Zheng et al. (2022)
Lonidamine	HK	Inhibits HK, particularly the mitochondrial-bound fraction, disrupting glycolysis	Phase II/III completed (other cancers); preclinical (HCC)	HCC, Prostate cancer, Lung cancer	Suboptimal efficacy as monotherapy, specific toxicity	Lei et al. (2023); Lu et al. (2024)
2-DG	HK	Competitively inhibits HK, blocking glucose phosphorylation	Phase I/II (other cancers); preclinical (HCC)	HCC, Non-Hodgkin’s lymphoma, Cervical cancer, GC	Poor selectivity, cardiotoxicity	Wang et al. (2015); Sasaki et al. (2021); Christensen et al. (2025)
PFK-158	6-phosphofructo-2-kinase/fructose-2,6-bisphosphatase 3 (PFKFB3)	Inhibits PFKFB3, reducing glycolytic flux	Phase I completed (other cancers)	Lung cancer; Pancreatic cancer (PC)	Suboptimal efficacy as monotherapy, dose-limiting toxicity	Thirusangu et al. (2022)
VDA-1275	HK2-VDAC1	Disrupts HK2 binding to mitochondrial VDAC1, reverses aerobic glycolysis	Preclinical; ready for Phase I	HCC, NSCLC, Prostate cancer, RCR	Developability challenges	Herzberg et al. (2024)
LY294002	PI3K/Akt, HK2, PKM2	Inhibits PI3K/Akt signaling, suppressing glycolytic gene expression	Preclinical	HCC, RCC, Differentiated thyroid carcinoma	Chemical instability	Huang et al. (2021); Li et al. (2024)
Linarin	PI3K/Akt/mTOR, HIF-1α	Suppresses PI3K/Akt/mTOR and HIF-1α pathways, downregulating glycolytic enzymes	Preclinical	HCC	Extremely low bioavailability	Zhang et al. (2024b); Wang et al. (2025a)
shikonin	PKM2	Inhibits PKM2, reducing pyruvate and lactate production	Preclinical	HCC, Ovarian cancer	Low bioavailability, poor targeting, hepatorenal toxicity	Chen et al. (2011); Zhang et al. (2024a)
Bay 11-7082	NF-κB, HK2, LDHA	Suppression of NF-κB downregulates HK2 and LDHA, abrogating lactate accumulation	Preclinical	GC, RCC	Cytotoxicity	Chen et al. (2014); Li et al. (2025)
α-Cyano-4-hydroxycinnamic acid	MCT1/MCT4	Blocks lactate efflux by inhibiting MCTs	Preclinical	BC, Glioblastoma, PC	Weak activity	Ferreira et al. (2020); Guan and Morris (2020)
10,058-F4	c-Myc	Disrupts c-Myc/Max interaction, thereby indirectly downregulating glycolysis	Preclinical	Acute myeloid leukemia, Neuroblastoma, PC	Weak activity, poor physicochemical properties, unstable *in vivo* metabolism	Zhang et al. (2015a); Broecker-Preuss et al. (2017)
KN93	Calcium/calmodulin-dependent protein kinase IV (CaMKIV)	Inhibits CaMKIV, interfering with calcium-dependent glycolytic regulation	Preclinical	Malignant mesothelioma	Poor water solubility, low bioavailability, unstable *in vivo* metabolism	Qi et al. (2019)
WZB117	GLUT1	Selectively inhibits GLUT1-mediated glucose uptake	Preclinical	HCC, lung cancer, BC	Poor solubility, off-target toxicity	Liu et al. (2012); Lu et al. (2021)
NaBu	HK2, PFK1, LDHA	Reduces the expression levels of HK2, PFK1, and LDHA	Preclinical	HCC	Poor pharmacokinetic, poor targeting	Yu et al. (2022)
DML	Lactylation	Inhibition of histone H3 lactylation	Preclinical	PLC	Insufficient activity of parent compound	Pan et al. (2022)
3-Bromopyruvate	HK2	Irreversibly inhibits HK2, disrupting glycolytic energy production	Preclinical	HCC, PC	Poor serum stability, systemic toxicity, short tumor retention time	Lee et al. (2017); Jia et al. (2021)
GSK2837808A	LDHA	Selectively inhibits LDHA, blocking lactate production	Preclinical	PC, Nasopharyngeal carcinoma, Melanoma	Extremely poor pharmacokinetics, poor solubility	Gupta et al. (2021); Qi et al. (2021); Zhang et al. (2026)

### 5.1 Inhibiting lactate production and transportation

Considering that tumor cells rely heavily on aerobic glycolysis as the main metabolic pathway for their function and that the formation of acidic TME from lactate-enriched extracellular tumor cells positively affects tumor development, proliferation, and metastasis, it is theoretically feasible to target the transport and production of glucose and lactate during aerobic glycolysis to treat HCC. Some results and other ongoing clinical studies have demonstrated that this strategy may be a novel and practical approach.

The first step of aerobic glycolysis is the translocation of glucose by GLUTs into tumor cells; therefore, targeting this transporter protein to deprive tumor cells of glucose can be referred to as the first step in inhibiting aerobic glycolysis. Commonly investigated drugs and monomers include silymarin, rhizopodophyllotoxin, quercetin and sodium-glucose linked transporter 2 (SGLT2) inhibitors and so on. Among them, silymarin (Zhan et al., 2011), rhizopodophyllotoxin, and quercetin are flavonoids extracted from natural products, which can be used as natural GLUT blockers to directly inhibit HCC growth, induce apoptosis in tumor cells or potentiate the toxic effects of anticancer drugs against tumor cells. In addition, studies have shown that SGLT2 inhibitors, currently used in treating type 2 diabetes mellitus, may have potential anticancer properties, as they cause tumor cell death by blocking the uptake of glucose by cancer cells. The SGLT2 inhibitor cagliflozin has been shown to blocks glucose uptake by HCC cells, thereby inhibiting cell proliferation and growth in a mouse liver tumor model. In human and mouse NAFLD-associated liver tumors, toglitazar reduced the degree of chronic inflammation and steatosis in the liver, thereby inhibiting and slowing down the precancerous stage of NAFLD-associated liver tumors (Obara et al., 2017).

In addition to glucose transport, targeting the rate-limiting enzymes in the glycolytic process is also an essential part of inhibiting aerobic glycolysis, including 2-deoxy-D-glucose (2-DG), dichloroacetate (DCA) and 3-bromopyruvate (3-BrPA). 2-DG is a glucose analog that competitively inhibits glucose uptake by GLUT1 and destroys HK early in the onset of glycolysis. 2-DG has been shown in *ex vivo* experimental studies to induce metabolic arrest and apoptosis in HCC cells (Zhang Y. et al., 2015). DCA can inhibit pyruvate dehydrogenase kinase (PDK) by promoting the conversion of pyruvate to acetyl coenzyme A, which ultimately reverses the glycolytic phenotype and converts the cellular metabolic pathway from glycolysis to oxidative phosphorylation and reduces lactate production. However, the efficacy of monotherapy with this drug cannot be determined, and treatment with a combination of other drugs is still required. For example, it can be observed in a mouse model of HCC that DCA treatment in combination with sorafenib effectively reduces sorafenib resistance (Shen et al., 2013). 3-BrPA is a metabolism inhibitor derived from halogenated derivatives of pyruvic acid, and it directly binds to and inhibits glyceraldehyde-3-phosphate dehydrogenase in radiolabeling studies, thereby inhibiting glycolysis and reducing ATP production, ultimately leading to apoptosis. Given 3-BrPA has the characteristics of total tumor coverage and high local drug concentration, it is frequently employed in image-guided intra-arterial therapy for patients with hepatocellular carcinoma. However, the appropriate dose should be determined when using it to prevent excessive doses from causing peripheral liver injury.

The completion of aerobic glycolysis is orchestrated by the generation and transport of lactate. therefore, it is hypothesized that blocking or inhibiting the expression of LDHA and MCT4 may reduce lactate levels and subsequently decrease PD-L1 lactylation, holding substantial therapeutic potential for HCC. LDHA is constitutively active within tumor cells, facilitating the conversion of pyruvate to lactate. Pharmacological inhibition or genetic ablation of LDHA essentially severs the metabolic flux at the source by abrogating lactate production, thereby suppressing malignant progression in various cancers (Xie et al., 2014; Chen D. et al., 2024). However, tumor cells exhibit profound metabolic plasticity. When the glycolytic pathway is inhibited, cells may initiate compensatory metabolic reprogramming to sustain bioenergetic and biosynthetic demands (Méndez-Lucas et al., 2020). Moreover, under physiological conditions, erythrocytes and skeletal muscle cells require LDHA to convert pyruvate into lactate to support normal physiological functions (Taha et al., 2026). Consequently, indiscriminate inhibition of LDHA expression may precipitate numerous off-target effects, exerting detrimental consequences on other lactate-dependent cells and tissues—a key factor that has considerably impeded the clinical advancement of such inhibitors. Accordingly, the specific ablation of tumor cell-associated LDHA may represent a promising direction for future targeted therapeutic strategies.

In contrast, strategies targeting MCT4 focus on blocking the critical channel responsible for lactate efflux from the intracellular to the extracellular compartment. Upon MCT4 inhibition, even with sustained intracellular lactate production, efficient efflux is impeded, leading to substantial lactate accumulation within the cell and subsequent intracellular acidosis. This, in turn, directly suppresses tumor cells through mechanisms including impairment of DNA repair, induction of mitochondrial dysfunction, and activation of apoptotic pathways. Studies have demonstrated that MCT4 inhibition correlates with inactivation of HIF-1α and downregulation of AKT pathway phosphorylation (Gao et al., 2015), further supporting MCT4 as a potential therapeutic target for HCC. More importantly, blockade of lactate efflux concurrently attenuates the acidification of the TME, thereby alleviating the immunosuppressive effects of an acidic TME and facilitating the restoration of antitumor immune activity. Leveraging this intracellular-extracellular cooperative mechanism, the combination of MCT4 inhibitors with immune checkpoint inhibitors has emerged as a prominent focus in current research on metabolism-immunity combination therapeutic strategies.

### 5.2 Modulating acidic TME

In addition, alongside investigations into the targeted inhibition of the aerobic glycolysis metabolic pathway, there is a burgeoning interest in the targeted modulation of the acidic TME via pharmacological agents. Notably, the administration of alkaline drugs or regulatory compounds via direct oral ingestion or localized injections has garnered significant attention. Experiments have demonstrated that oral administration of alkaline buffers can neutralize TME acidity, increasing pH and inhibiting the metastatic tendency of tumors (Ibrahim Hashim et al., 2011). Moreover, mTOR, in combination with aqueous NaHCO3, has shown superior anticancer effects compared with mTOR alone (Faes et al., 2016). Demethylzeylasteral (DML) is a triterpenoid anti-tumor compound, and it has been confirmed by relevant biological tests that DML can inhibit H3 histone lactylation and thus inhibit HCC stem cell-induced tumorigenicity, which side-steps the developmental potential of DML for the treatment of HCC(Pan et al., 2022). However, as a natural product, DML currently lacks systematic evaluation data on key drug-like properties such as stability, *in vivo* metabolic profile, and bioavailability. Existing studies are predominantly limited to *in vitro* cellular experiments, with only a few having progressed to the stage of *in vivo* animal studies.

In addition to oral targeted drugs, TACE and HAIC are now widely used to treat patients with unresectable tumors. Although the clinical efficacy is better, it is still difficult to completely block tumor development, and recurrence is easier after surgery. Therefore, interventionalists at home and abroad have begun to use TACE together with catheter infusion of local regulatory drugs such as anti-angiogenic or immunity-enhancing drugs (Geng - fei et al., 2015; Dalin, 2017). Studies on precise regulation of local acid–base balance in tumor cells have been continuously pursued to inhibit tumor proliferation and metastasis, and to treat unresectable HCC. This strategy offers specific and safer therapeutic efficacy. Local infusion of bicarbonate in post-TACE patients showed that the TACE group exhibited better anticancer activity and more prolonged survival than non-treated patients (Chao et al., 2016). However, this local infusion therapy is currently only uesd for local tumor control during the observation period and cannot predict or guarantee prognosis and overall survival. Nonetheless, it is still expected to become a new generation of accurate and minimally invasive treatment.

### 5.3 Combination of targeted agents and metabolic interventions

Sorafenib and lenvatinib, molecularly targeted multikinase inhibitors, are currently approved as first-line targeted agents for the treatment of advanced HCC. In recent years, second-line agents such as regorafenib, cabozantinib, and rimodulizumab have also been gradually introduced into clinical practice. However, it is still unavoidable that immune resistance occurs in HCC patients in the late stage of treatment; therefore, reducing or even completely blocking immune resistance, improving immune response, or screening patients with good immune response is a serious challenge for treatment. The mechanisms underlying chemotherapy drug resistance are highly complex, potentially involving metabolic heterogeneity within TME cellular components such as cancer-associated fibroblasts (CAFs) and TAMs (Eun et al., 2023), long non-coding RNAs (Gao et al., 2023), and ferroptosis (Tang et al., 2023). Specifically, CAFs enhance glycolysis via GLUT1 and PKM2 expression, actively repelling cytotoxic T-cell infiltration through CXCL1 expression, thereby directly inducing chemotherapy resistance (Broz et al., 2024; He et al., 2025). Conversely, TAMs modulate drug effects on tumor immune microenvironments according to differing M1/M2 metabolic phenotypes (Wang X. et al., 2025).

Inhibition of intracellular lactic acidification attenuates the resistance of HCC cells to sorafenib. The multi-targeted role played within the bioenergetic spectrum, as detected after extraction of the ethyl acetate extract of the edible plant sea fennel, can be directly demonstrated by the fact that this extract increased the sensitivity of HCC to sorafenib by decreasing lactate fermentation (Gnocchi et al., 2023). Given that forkhead box O6 (FOXO6) is upregulated in HCC and promotes tumor cell proliferation by regulating glycolysis via the PI3K/Akt signaling pathway, multilevel experimental evidence has demonstrated that treatment with the inhibitor LY294002 reduces FOXO6 expression, thereby suppressing glycolysis, decreasing lactate production in HCC cells, and enhancing their resistance to the chemotherapeutic agent paclitaxel (Yu et al., 2020). However, owing to its extremely poor pharmacokinetic profile and chemical instability, its advancement into clinical trials has been precluded.

The influence of gut microbiota and their metabolites on aerobic glycolysis can similarly suppress immune response of HCC to chemotherapeutic agents. Consequently, pharmacological modulation of gut probiotics, specific inhibition of microbial metabolites, or fecal microbiota transplantation may concurrently regulate both glycolysis and immune responses, offering novel strategies for combined immunotherapy (Jia et al., 2026). *In vitro* and *in vivo* experiments confirmed that NaBu decreases the expression levels of HK2, PFK1, and LDHA in a dose-dependent manner, thereby decreasing lactate production. This directly inhibits aerobic glycolysis, suppressing proliferation and inducing apoptosis of HCC cell. The study further demonstrated that NaBu enhances the anti-HCC efficacy of sorafenib both *in vitro* and *in vivo* through the aforementioned mechanisms (Yu et al., 2022). Although NaBu is occasionally employed in an adjuvant setting, it is not classified as a clinical anticancer agent. Owing to its poor pharmacokinetic properties and lack of targeting, which confer a risk of off-target effects, it is more frequently utilized as a tool for mechanistic investigations.

In summary, future therapeutic strategies must move beyond the single-target paradigm toward multi-target metabolic interventions. Such an approach should integrate modulation of the glycolytic pathway with strategies aimed at remodeling the TME, alongside longitudinal monitoring of metabolic activity. Only through such a comprehensive strategy can resistance driven by tumor heterogeneity be effectively circumvented, thereby improving treatment outcomes and prognosis for patients with HCC. Nevertheless, despite this therapeutic rationale, the safety, efficacy, and drugability of inhibitors targeting the aerobic glycolysis–lactate–acidic TME axis remain predominantly confined to the preclinical stage. At present, these inhibitors are primarily employed as tools in fundamental research to dissect relevant biological pathways, with only a limited number having progressed to clinical trials. This disparity highlights a substantial gap in clinical translation within the field of HCC therapy, while concurrently delineating promising avenues for future drug development.

## 6 Challenges and Perspectives

Aerobic glycolysis is a hallmark metabolic feature of HCC development and plays an essential role in HCC development, proliferation, metastasis, and immune resistance. The large amount of lactate produced through various biological enzymes and signaling pathways after the high activation of aerobic glycolysis is equally crucial for further activation of the glycolytic pathway and promotion of tumor development for the following reasons. First, the energy supply efficiency of ATP produced by the aerobic glycolytic pathway is much higher than that of oxidative phosphorylation. Secondly, the acidic TME formed after the enrichment of lactate inhibits the growth and development of normal cells and normal cells in other tissues of the body through the inhibition of oxygen and the robbing of nutrients, and so on. Third, acidic TME is undoubtedly the most suitable external environment for the survival and development of HCC cells.

To address this complex mechanism, novel and effective research and diagnostic strategies have been proposed and validated, such as targeting biological proteins that play a role in the glycolysis pathway and related pathways or gene sequences to regulate lactate production and transport, alkalizing the TME by oral or local infusion of alkaline buffer. It may also enhance the body’s immune responsiveness by addressing metabolic heterogeneity within TME, thereby mitigating resistance to targeted therapies. By altering the composition and proportions of gut microbiota, it can directly or indirectly inhibit aerobic glycolysis through the gut-liver axis, thereby increasing sensitivity to chemotherapeutic agents. Understanding the mechanism of action of different programs or drugs will help to select accurate and effective therapeutic programs for various groups of patients with varying sensitivities to drugs in subsequent experiments and clinics.

Despite substantial progress in understanding the mechanisms of aerobic glycolysis in HCC, several major challenges continue to impede clinical translation. A primary hurdle is the considerable inter-and intra-tumoral metabolic heterogeneity of HCC. Different regions of the same tumor, or different patients, may exhibit divergent glycolytic dependency, leading to suboptimal efficacy of single-agent glycolytic inhibitors (An and Li, 2025). To address this heterogeneity, patient stratification using biomarkers can help identify those with glycolysis-addicted tumors. Moreover, combination regimens targeting multiple metabolic nodes, such as glycolysis together with glutaminolysis, may overcome clonal heterogeneity and improve clinical outcomes.

Another critical challenge is the remarkable metabolic plasticity of HCC cells. When glycolysis is inhibited, tumor cells rapidly adapt by upregulating alternative energy pathways (Tufail et al., 2024), such as glutamine metabolism, fatty acid oxidation, or mitochondrial oxidative phosphorylation. This adaptive escape allows them to circumvent single-target blockade. To counter this plasticity, dual or sequential inhibition of both glycolysis and the most common escape pathways has shown synergistic effects in preclinical models. For instance, combining an HK2 inhibitor with a glutaminase inhibitor, or employing intermittent dosing schedules, may delay the emergence of adaptive resistance.

A third major obstacle is off-target toxicity. Many glycolytic enzymes are also essential in normal tissues. Non-selective inhibition can cause dose-limiting toxicities such as cardiac dysfunction or neurotoxicity, compromising patient quality of life and treatment adherence. To mitigate this, concrete solutions include targeting tumor-specific isoforms, such as HK2 instead of HK1 (Garcia et al., 2019), using prodrugs activated only in the hypoxic/acidic TME (Nazli et al., 2024; Liu et al., 2025), and employing locoregional delivery such as hepatic artery infusion or nanoparticles to concentrate the drug in liver tumors while limiting systemic exposure (Pourbaghi et al., 2023). Integrating these approaches, along with biomarker-driven patient selection and rational combination regimens, can systematically address the translational hurdles of heterogeneity, plasticity, and toxicity, paving the way for safer and more effective metabolism-targeted therapies for HCC.

In the next 3–5 years, research on metabolic targeted therapies for HCC should establish a clear pathway encompassing prediction, analysis, and intervention.

A key priority for prediction and patient stratification is the development of cost-effective and minimally invasive biomarkers—such as serum lactate levels, expression of glycolytic enzymes, and gut microbiota signatures—to identify HCC patients most likely to benefit from glycolysis-targeted or lactate-modulating therapies. Leveraging single-cell multi-omics technologies will further delineate the cellular heterogeneity of metabolic–immune crosstalk within TME, thereby refining these predictive tools. In parallel, for dynamic analysis and treatment monitoring, metabolic imaging-guided approaches—including hyperpolarized MRI and novel lactate-sensitive tracers—could enable real-time, quantitative assessment of glycolytic activity and early evaluation of therapeutic response. These imaging modalities may be integrated into clinical trials as pharmacodynamic biomarkers.

Regarding intervention, the most feasible near-term strategy is the combination of metabolic inhibitors (e.g., MCT4 inhibitors) with immune checkpoint blockade, given that preclinical evidence already supports their synergistic potential. In parallel, precision microbiota interventions targeting the gut-liver axis could serve as an adjunct to enhance immunotherapy efficacy. Collectively, this multidimensional reprogramming of the TME—through biomarker-driven patient selection, imaging-guided response monitoring, and rationally designed combination regimens—holds promise for improving overall therapeutic outcomes in HCC within the coming years.

## Glossary

PLC: primary liver cancer
HCC: Hepatocellular carcinoma
TME: tumor microenvironment
ATP: adenosine triphosphate
GLUTs: glucose transporters
Glucose-6-P: glucose-6-phosphate
HK2: hexokinase 2
Fructose-6-P: fructose-6-phosphate
Fructose-1,6-BP: fructose-1,6-bisphosphate
PFK1: phosphofructokinase-1
PKM2: pyruvate kinase M2
PDH: pyruvate dehydrogenase
LDH: lactate dehydrogenase
MCTs: monocarboxylate transporters
GC: gastric cancer
NSCLC: non-small-cell lung cancer
BC: breast cancer
mtDNA: Mitochondrial DNA
mTOR: Mammalian target of rapamycin
HIF-1α: hypoxia-inducible factor-1α
Treg cells: regulatory T cells
Kla: histone lysine lactylation
GPR81: G-protein-coupled receptor 81
ASICs: acid-sensing ion channels
PD-L1: programmed death-ligand 1
NaBu: sodium butyrate
**TAM**s: tumor-associated macrophages
ACE2: angiotensin-converting enzyme 2
TACE: transcatheter arterial chemoembolization
HAIC: hepatic artery infusion chemotherapy
SGLT2: sodium-glucose linked transporter 2
2-DG: 2-deoxy-D-glucose
DCA: dichloroacetate
3-BrPA: 3-bromopyruvate
PDK: pyruvate dehydrogenase kinase
DML: Demethylzeylasteral
CAFs: cancer-associated fibroblasts
FOXO6: forkhead box O6
CRC: Colorectal Cancer
RCC: Renal Cell Carcinoma
CaMKIV: Calcium/calmodulin-dependent protein kinase IV
PFKFB3: 6-phosphofructo-2-kinase/fructose-2,6-bisphosphatase 3
PC: Pancreatic cancer
